# Extraction of Camphor Tree Essential Oil by Steam Distillation and Supercritical CO_2_ Extraction

**DOI:** 10.3390/molecules27175385

**Published:** 2022-08-24

**Authors:** Huangxian Zhang, Ting Huang, Xiaoning Liao, Yaohong Zhou, Shangxing Chen, Jing Chen, Wanming Xiong

**Affiliations:** 1 College of Chemistry and Materials, Jiangxi Agricultural University, Nanchang 330045, China; 2 National Forestry and Grassland Bureau Woody Spice (East China) Engineering Technology Research Center, Jiangxi Agricultural University, Nanchang 330045, China; 3 School of Information and Engineering, Jiangxi Agricultural University, Nanchang 330045, China

**Keywords:** steam distillation, supercritical CO_2_ extraction, camphor tree, essential oil, terpenoids

## Abstract

The essential oil extracted from *Cinnamomum camphora* leaves is a mixture of volatile compounds, mainly terpenes, and is widely used in medicine, perfume and chemical industries. In this study, the extraction processes of essential oil from *Cinnamomum camphora* leaves by steam distillation and supercritical CO_2_ extraction were summarized and compared, and the camphor tree essential oil was detected by GC/MS. The extraction rate of essential oil extracted by steam distillation is less than 0.5%, while that of supercritical CO_2_ extraction is 4.63% at 25 MPa, 45 °C and 2.5 h. GC/MS identified 21 and 42 compounds, respectively. The content of alcohols in the essential oil is more than 35%, and that of terpenoids is more than 80%. The steam extraction method can extract volatile substances with a low boiling point and more esters and epoxides; The supercritical method is suitable for extracting weak polar substances with a high alcohol content. Supercritical CO_2_ extraction can selectively extract essential oil components and effectively prevent oxidation and the escape of heat sensitive substances.

## 1. Introduction

An essential oil is a kind of aromatic mixture with a low molecular weight and high volatility, produced in the process of plant physiological metabolism. Plant essential oils and their derivatives are important raw materials in the chemical industry and have an important economic value, so they are also called liquid gold [1]. At present, more than 3000 kinds of essential oils have been found, and more than 1340 species have antibacterial activity, of which about 300 varieties of essential oils have been developed and utilized [2,3]. Therefore, an in-depth study of essential oils is quite promising.

Camphor tree, also known as *Cinnamomum camphora*, is a special tree species in China. It is an important raw material for condiments, spices, flavor, pharmaceuticals and chemical industries [4]. Camphor tree essential oil contains many chemical components, such as linalool, eucalyptus, camphor, neroli tertiary alcohol, etc. Camphor tree essential oil has a wide range of biological functions, including antibacterial, antioxidant, antifungal, anti-inflammatory, insecticidal and repellent properties [5,6,7]. For example, linalool has antibacterial, antiviral and sedative effects, and the minimum bactericidal concentration (MBC) is 200 μL/L [8]. Moreover, it also can be used as a strategy for the treatment of neurodegenerative diseases because of a potential neuroprotective effect [9]. Eucalyptus has been used in the preparation of oral condiments as food additives and drug research preparations. Because of its anti-inflammatory effect, it can also improve the lung function of asthmatic rats by intramedullary injection [10]. Camphor contributes to analgesia and reduces inflammation [11], has a certain protective effect in mosquito repellents, and has a strong inhibitory effect on mold growth [12]. Nerol is a sesquiterpene with natural membrane activity, which has the advantages of antitumor, antibacterial, antifungal and antiparasitic activities [13].

There are many extraction methods for the extraction of camphor tree essential oil. Different extraction methods have remarkable differences in the yield of essential oil, and have a significant impact on the chemical components extracted [14]. Extraction methods mainly include steam extraction, organic solvent extraction, ionic liquid combined with microwave-assisted extraction, solvent-free microwave extraction, cold-pressed extraction, supercritical fluid method, subcritical CO_2_ extraction, ultrasound-assisted extraction, microwave-assisted extraction, etc. [15,16,17]. In order to maintain the biological activity of camphor tree essential oil, it is necessary to find a suitable extraction technology [18]. The most widely used supercritical CO_2_ method is non-toxic, relatively inert, and non-flammable; the extraction effect can be improved by the tuning of pressure and temperature [19]. Due to supercritical CO_2_ possessing a higher diffusivity, solubility, and selectivity than regular fluid extraction [20], it has been tested and applied in the extraction of compounds from various types of plant parts [21,22].

In this paper, the essential oil of camphor tree leaves was extracted using both steam distillation and supercritical carbon dioxide extraction. The main chemical components and the contents of the extracted oils were analyzed using GC-MS. The extraction results of the two methods were compared and the advantages and disadvantages of each method were analyzed, so as to provide a reference for the in-depth study of plant essential oils in the future.

## 2. Results and Discussion

### 2.1. Steam Distillation

The extraction rate of essential oils obtained from the data after anhydrous treatment by steam distillation is shown in Table 1. The mass of raw material (m_1_) weighed in each experiment was 100–200 g, and the mass of essential oil (m_2_) obtained was about 0.5 g.

There was a difference in the yield of essential oil from the three materials: 0.480% (flower), >0.326% (leaf), and >0.212% (stem), which was also consistent with the distribution law of essential oil. Compared with the data values obtained in other literatures [23,24,25,26], the extraction rates obtained in this experiment were lower. In addition, the average extraction rates of the three samples were below 0.5%, which may be because the camphor tree raw materials were all fresh samples with an excessive water content. On the other hand, water-insoluble essential oils could be collected by this method, but the components in the aqueous phase were lost.

### 2.2. Supercritical CO_2_ Extraction

#### 2.2.1. Single Factor Experiment

The extraction selectivity and yield depended on the SC-CO_2_ density which was regulated by the operation parameters. Herein, the key factors included temperature, pressure and time, and their effects on the extractions were as follows.

**Figure 1:**
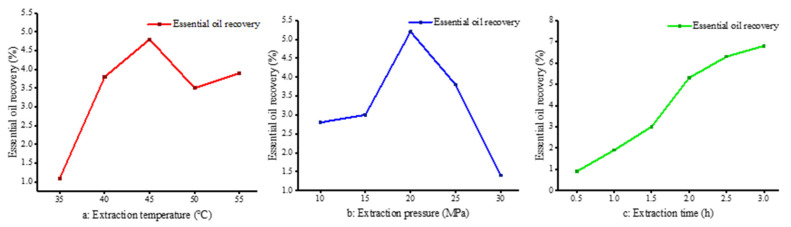
Effect of different conditions on extraction rate. (**a**: Extraction temperature, P = 20 MPa, Tm = 2 h; **b**: Extraction pressure, T = 45 °C, T_m_ = 2 h; **c**: Extraction time, T = 45 °C, P = 20 MPa).

The influence of extraction temperature on supercritical fluid is complex. On the one hand, temperature has an effect on the concentration of carbon dioxide. High temperature will reduce the density of carbon dioxide fluid, weaken the dissolution effect, reduce the solubility of materials, thus reducing the yield. On the other hand, under a certain pressure, the temperature rises, the volatility of the extraction solute and the thermal motion increases the opportunity of intermolecular association, thereby improving the extraction rate. Therefore, an increase in temperature facilitates the diffusion of the solute, but may also reduce its solubility; the extraction rate depends on which state prevails at a given temperature. The extraction yield of essential oil at different temperatures (35 °C, 40 °C, 45 °C, 50 °C and 55 °C) was studied at a set pressure (20 MPa) and extraction time (2 h). The highest extraction rate was about 5% at 45 °C. Below 45 °C, the extraction rate increased with an increase in temperature. From 45 to 55 °C, the extraction rate decreased slightly and tended to be stable. The extraction rate of essential oil obtained from the sorted data is shown in Figure 1a.

In this section, the yield of essential oil under different extraction pressures (10 MPa, 15 MPa, 20 MPa, 25 MPa and 30 MPa) was studied at a set temperature (45 °C) and extraction time (2 h) conditions. The extraction rate was highest at 20 MPa, which was nearly 5%. When the pressure was lower than 20 MPa, the higher the pressure, the higher the extraction rate. The growth rate was relatively rapid, from 15 MPa to 20 MPa. When the extraction pressure exceeded 20 MPa, the extraction rate decreased significantly. The results are shown in Figure 1b. The extraction pressure had a significant effect on the extraction rate. On the one hand, increasing the pressure not only increases the density of carbon dioxide, but it also reduces the intermolecular distance and improves the mass transfer efficiency. Pure camphor is a solid at temperatures of 304 to 354 K; nonetheless, when in contact with carbon dioxide, it dissolves readily and reaches phase equilibrium [27]. As the pressure was increased from 15 MPa to 30 MPa, more oxygenated compounds, such as oxidized sesquiterpenes, were present, which is also consistent with the findings of others [28] regarding the higher oxygen content in extracts at elevated SC-CO_2_ pressures. This further illustrates that changes in pressure cause significant changes in the density of CO_2_, which in turn causes changes in the solubility of the substance to be extracted for the purpose of selective extraction. On the other hand, these two factors are not linear. When the pressure increases to a certain extent, the dissolution rate increases slowly, thus affecting the extraction rate. In addition, the higher the supercritical pressure, the greater the density of carbon dioxide, the more complex the extract, and the worse the selectivity [29].

The influence of extraction time cannot be ignored because the extraction effect is poor when the extraction time is too short. Similarly, if the time is too long, it not only reduces the production efficiency of the equipment, but also wastes energy and manpower. Therefore, it is necessary to determine the optimal extraction time to achieve the best extraction rate. As can be seen from Figure 1c, the extraction rate increased rapidly at the beginning of extraction, slowed down after the extraction time of 2.0 h, and barely increased after 3.0 h. This is because at the initial stage of extraction, the content of essential oil in camphor leaves is larger, so the extraction rate increases rapidly; with the extension of time, the essential oil content in camphor leaves gradually decreases, leading to reduced increases in extraction rates.

#### 2.2.2. Orthogonal Experiment

In order to make the extraction process more reasonable and scientific, an orthogonal experiment was carried out on the basis of a single factor experiment. The three important factors of extraction time, extraction pressure and extraction temperature were tested. The experimental results are shown in the following Table 2.

The orthogonal test results showed that the factors affecting the extraction rate were extraction time > extraction pressure > extraction temperature. The optimum combination was an extraction pressure of 25 MPa, an extraction temperature of 45 °C, and an extraction time of 2.5 h, with a yield approaching 4.63%.

### 2.3. Analysis of Essential Oil Component

According to the total ion chromatography analysis of the E-oil, the peak of the substances was mainly concentrated after 10 min. Twenty-one compounds were identified from the essential oils of leaves. The detailed result is summarized in Table 3a. The main chemical components of the essential oil were neroli tertiary alcohol and caryophyllene oxide, while the contents of linalool and camphor were not significant.

**Figure 2:**
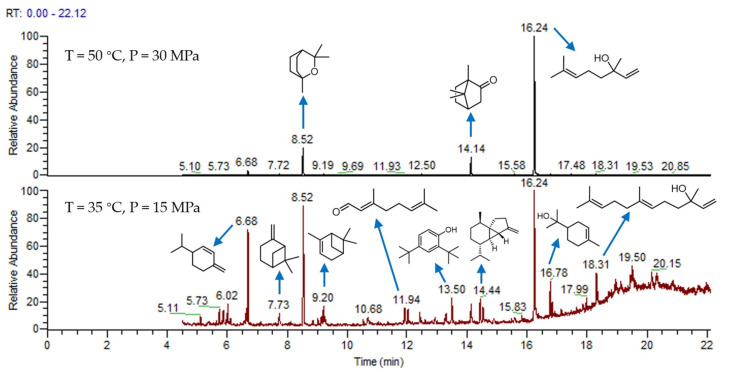
GC-MS of camphor tree leaves essential oil.

For comparison, two cases of supercritical CO_2_ extraction under two extreme conditions were selected, and the essential oil was analyzed by GC-MS, as shown in Figure 2. The main substances were concentrated after 6 min, and 26 compounds were identified in the essential oil. The results are shown in Table 3b,c. The total areas of b and c are 99.77 and 98.5%, respectively. The results show that supercritical CO_2_ extraction can selectively extract most of the essential oil as much as possible. Under the condition of 50 °C and 30 MPa, the components of the essential oil are selective, among which linalool is the majority, camphor and α-pinene are the minority. Understandably, the main extract components have the characteristics of a high boiling point and a relatively high molecular weight. At 35 °C and 15 MPa, the components of the essential oil are complex. The main components are α-terpineol, cubebene and β-phellandrene, which is a compound with a low boiling point and a relatively low molecular weight.

The essential oil composition and peak area percentages of *Cinnamomum camphora* leaves under different conditions and methods, was ordered and summarized, as shown in Table 3:

Forty-two compounds were identified from the essential oil of camphor tree leaves, as shown in Table 3. Camphor tree essential oil contains a large amount of terpenoids. Among the 42 identified compounds, 29 are terpenoids, including 9 monoterpenoids and 20 sesquiterpenoids, accounting for 82.38% of the total essential oil content. The results of GC-MS analysis showed that the compositions of the essential oils extracted by the two methods were very different. The main components were obviously different; only four substances were common to the two methods, and their content was about 30%. The content of hydrocarbons, alcohols and oxides varied greatly. More oxides were extracted by the steam method, while more alcohols were extracted by the supercritical method. The yield of essential oil by steam distillation is low, extracting low molecular, water insoluble substances with low boiling points, such as nerolidol, β-eudesmene and caryophyllene oxide. Supercritical CO_2_ extraction has a good extraction yield and component integrity, which can effectively prevent oxidation and the escape of heat sensitive substances, but it is difficult to extract substances with relatively high molecular weights. When supercritical CO_2_ extraction is used, more substances are extracted at low temperature and pressure, including 26 extracted substances; The number and types of substances extracted under high temperature and pressure decreased significantly, with only 13 substances extracted. More hydrocarbons were extracted at 35 °C and 15 MPa (50.03% > 18.58%), such as β-phellandrene, α-pinene and cubebene; More alcohols were extracted at 50 °C and 30 MPa (68.95% > 33.56%), such as linalool. It can be seen that a low temperature and pressure is conducive to the extraction of substances with low boiling points and relatively low molecular weights. By increasing the temperature and pressure, the fluid characteristics can be changed, so as to mainly extract substances with high boiling points and relatively high molecular weights, thus laying a foundation for supercritical purification.

**Figure 3:**
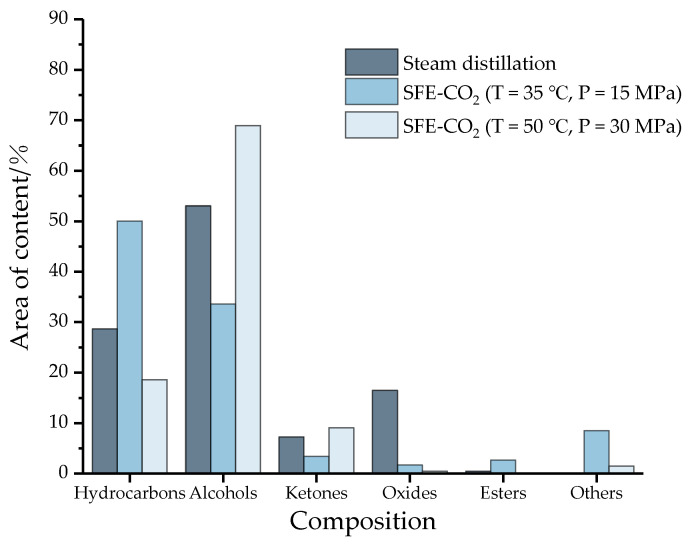
The chemical composition and area content of essential oil under three conditions (SFE-CO_2_: Supercritical carbon dioxide extraction; Others: ethers, aldehydes, phenols and acids).

According to the conventional classification of compounds, the 42 components of the essential oil (18 hydrocarbons, 11 alcohols, 2 esters, 4 ketones, 3 oxides, 1 ether, 1 aldehyde, 1 phenol and 1 acid) can be divided into 6 categories: hydrocarbons, alcohols, ketones, oxides, ethers and others. The results are shown in Figure 3.

Obviously, hydrocarbons and alcohols are the main components of essential oils. Among them, the alcohol content of the three essential oil components exceeds 33%, and the content of hydrocarbons exceeds 18%. However, the content of oxides and ketones is less, and the content of esters is close to zero. The main components of essential oils are mainly volatile terpenes and olefins, and simple terpenes with special aroma components [30], which may be the main source of the aroma of camphor tree essential oil. In addition, compared with the supercritical method, the content of oxides (e.g., caryophyllene oxide) in the distillation process is higher, approaching 20%, while substances with poor water solubility or thermal stability, such as aldehydes, phenols, and acids (others) are lost. The distribution of this species is consistent with the literature values [31,32]. In conclusion, there are significant differences in the composition of essential oils under different conditions in water vapor distillation and supercritical carbon dioxide extraction, and even in different supercritical methods.

### 2.4. Comparative Analysis of These Two Different Methods

#### 2.4.1. Steam Distillation

The water vapor distillation method is most commonly used to extract essential oils, using water as solvent. This method is more environmentally friendly. In the process of extraction and steam distillation, the temperature is high, the extraction time is long, the system is open, and the two steps are combined into one at the same time, thus reducing the trial process, shortening the analysis time, saving the extraction solvent, simplifying equipment and facilitating the operation [33,34]. Although the extraction rate is low, the purity of essential oil is high. Many substances are typical components of camphor tree essential oil, which can be further fractionated or purified by distillation. Meanwhile, high temperatures may lead to the volatilization or oxidation of allergic components in essential oils. This also occurred during the extraction of the volatile oil of Ligustrum quihoni by Wang et al. [35]. Only insoluble essential oils can be collected by this method, and the loss of water-soluble components leads to a low extraction rate. The extracted essential oil is still a multi-component composite essential oil, which is difficult to extract selectively, thereby affecting levels of purity, and the components of the collected essential oil are incomplete. In addition to using other separation techniques, obtaining purer essential oils may be more effective using other varieties of camphor trees.

#### 2.4.2. Supercritical CO_2_ Extraction

Supercritical carbon dioxide extraction technology uses fluid carbon dioxide as the extractant to extract effective components from plants, so as to maintain the purity and active components of essential oils to the greatest extent. Carbon dioxide, as an inert gas, does not have a chemical reaction in the extraction process, is safe and environmentally friendly. Essential oil components can be selectively extracted and further purified by selecting the appropriate temperature, pressure and extraction time.

Through experimental research, the extraction rate of supercritical carbon dioxide is found to be significantly higher than that of the conventional steam method, and it is also more advantageous in terms of time [36]; the extraction rate can be increased by more than two times, while the extraction time can be reduced by more than half. This is consistent with the results of extracting essential oil from firenut leaves [37], evening primrose [38] and patchouli [39]. This may be because, in addition to essential oils, some substances such as *n*-docosane, stearic acid and ergosterol can be extracted. Of course, pigments can also be extracted, so the purity of essential oil is not good enough. Some experiments [19] also show that supercritical extraction has a very key advantage in that its extraction temperature and pressure are highly adjustable; there are obvious differences in material types and the main components under different extraction conditions, which lays a foundation for selective extraction, meaning that the selective extraction of essential oil components can be tested by changing the extraction conditions. For example, with pressures from 15 MPa to 30 MPa, the percentages of major compounds such as linalool and camphor varied (from 19.81 to 66.42% and from 3.39 to 9.05%, respectively). However, the percentages of β-phellandrene, α-terpineol and α-pinene decreased significantly at higher pressures. Similar conclusions were reached by Moldão-Martins et al. [40], who described a decrease in monoterpene content with increasing pressure.

The extraction method of essential oil should be considered according to the chemical structure of the sample, required yield, required purity, production cost, economic benefit and other comprehensive factors. At the same time, the method used must be tested repeatedly to find the most suitable conditions. The data, conditions and experience obtained in this experiment are expected to provide powerful assistance to other relevant researchers.

## 3. Materials and Methods

### 3.1. Materials

Camphor tree materials involved in the study were randomly picked from the campus of Jiangxi Agricultural University, and the plant growth was good. After the samples were cleaned, they were crushed using a high speed universal pulverizer (FW100, Tianjin, China) and stored for following experiments. The supercritical fluid extraction device (HA121-50-01), with a 1000 mL stainless steel extraction vessel, was designed by Nantong Huaan (Jiangsu, China), and it was used to divide Camphor tree leaves into SC-CO_2_ soluble fraction (Essential oil, E-oil) and SC-CO_2_ insoluble fraction (residue).

### 3.2. Extraction Experiments of Camphor Tree Essential Oil

#### 3.2.1. Steam Distillation Extraction

The process of steam distillation extraction refers to method A for the determination of essential oil in Appendix XD of The Chinese Pharmacopoeia 2015 edition [41]. Camphor tree raw materials of a certain mass (m_1_) and 600 mL distilled water were added into a 1000 mL round bottom flask, and the essential oil collection device was assembled after mixing evenly. The extraction was carried out by heating with water vapor for 6 h and stopped when the yield of product no longer increased. The essential oil was cooled and collected, and dried with an appropriate amount of anhydrous sodium sulfate, then the oil phase was separated out and weighed (m_2_). The experiment was repeated three times and the average value taken. Finally, the E-oil was analyzed with GC or GC-MS (sealed and stored in cold storage away from light, if necessary). The extraction rate calculation formula was:

Extraction rate = (m_2_/m_1_) × 100% (1)

#### 3.2.2. Supercritical CO_2_ Extraction

**Figure 4:**
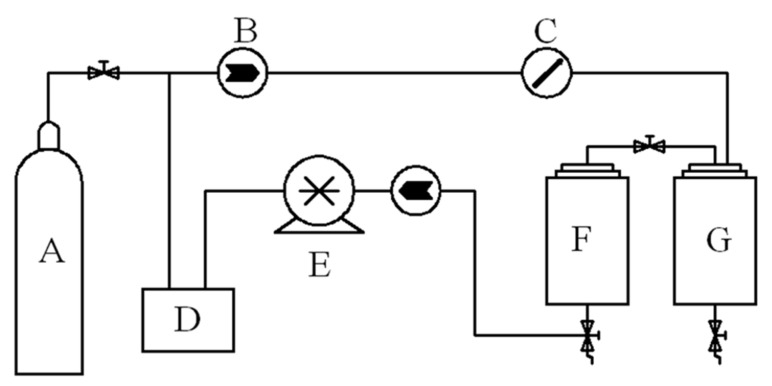
Schematic diagram of experimental apparatus. A: CO_2_ cylinder; B: check valve; C: flow meter; D: cold liquid circulator; E: piston pump; F: extraction vessel (1 L and 0.55 m height); G: separation vessel (0.6 L).

For all experiments of this section, the extraction vessel was packed with 60 g of camphor tree leaves. The extraction tests using supercritical CO_2_ were carried out using the equipment and flow diagram, as shown in Figure 4, and as follows: Carbon dioxide from the cylinder passed through a precooler, a positive displacement pump and a preheater before it entered the bottom of the extraction vessel. The extraction vessel was maintained at a predefined pressure (10–30 MPa) and temperature (35–55 °C). E-oil was recovered from the loaded solvent stream, expanded to system pressure (ca. 5 MPa) in the separation vessel. The flow of SC-CO_2_ was controlled by check valves and measured by a gas flow meter with an accuracy of ±0.01 kg/h. A variable frequency piston pump controlled the pressure in the vessel to an accuracy of ±0.1 bar. The extraction rate was calculated using the formula:

Extraction rate = m (essential oil)/m (camphor tree leaves) × 100%(2)

### 3.3. Analysis Methods

Gas chromatography-mass spectrometry (GC-MS-QP2010) was used to detect the components of the E-oil in the injected sample. Half a milliliter (0.5 mL) of camphor essential oil, extracted by water vapor distillation and supercritical CO_2_ fluid extraction, respectively, was weighed and methanol then added to fix the volume as the test solution in a 5 mL volumetric flask, 0.2 μL being taken for GC-MS analysis. Column conditions: Quartz capillary column (30 m × 0.25 mm, 0.25 μm); Carrier gas was high-purity helium, and flow rate was 1.0 mL/min; Shunt ratio was 40:1; Inlet temperature was 250 °C; The programmed temperature was set with an initial column temperature of 70 °C, and the temperature was maintained for 4.0 min. The temperature was raised to 250 °C at 4 °C/min, and then maintained for 5 min. Mass spectrum conditions: electron bombardment (EI) electron source, electron energy was 70 eV, the scanning quality range was 30 to 550 amu. The identification of the essential oil compounds from the analysis was as achieved by searching the NIST library of standard spectra and literature. The relative percentages of each component were calculated using the peak area normalization method. The component percentages were calculated as average values from duplicate GC-MS analyses of all the extracts.

## 4. Conclusions

The extraction of essential oil from camphor tree leaves by steam distillation and supercritical CO_2_ extraction was studied in this work. The results showed that the extraction rate of essential oil by steam distillation was less than 0.5%, while it was 4.63% for supercritical CO_2_ extraction. A total of 42 compounds were identified by GC/MS. The steam extraction method can extract volatile substances with low boiling points and more esters and epoxides; The supercritical method is suitable for extracting weak polar substances with a high alcohol content.

The components of the essential oil can be selectively extracted using the supercritical CO_2_ process with the following results: At 50 °C and 30 MPa, the main components are high boiling point and relatively high molecular weight components; while at 35 °C and 15 MPa, the main components of the essential oil are low boiling point and relatively low molecular weight components. The total content of terpenoids in the two methods is about 80%, which are the main contributors to the aroma of camphor tree essential oil. The extracted Camphor, Linalool and Nerolidol have important medical and biological uses, but the extracted yield is low, and subsequent purification is needed before application.

## Figures and Tables

**Table 1:** Experimental results of extraction of camphor tree leaf, flower and stem by steam distillation.

Material	m_1_ (g)	m_2_ (g)	Extraction Rate (%)
Leaves	199.93	0.65 ± 0.05	0.326 ± 0.026
Flowers	115.71	0.56 ± 0.07	0.480 ± 0.064
Stems	131.90	0.28 ± 0.04	0.212 ± 0.031

m_1_: Mass of raw material; m_2_: Mass of extracted essential oil. The extraction was carried out by heating with water vapor for 6 h. The experiment was repeated three times and the average value taken.

**Table 2:** Orthogonal test results of supercritical CO_2_ extraction.

	Factors	A	B	C	Mass (g)
Experimental Number					
1		20 MPa	40 °C	0.5 h	1.40
2		20 MPa	45 °C	1.5 h	3.40
3		20 MPa	50 °C	2.5 h	2.85
4		25 MPa	40 °C	1.5 h	3.18
5		25 MPa	45 °C	2.5 h	4.63
6		25 MPa	50 °C	0.5 h	1.73
7		30 MPa	40 °C	2.5 h	2.48
8		30 MPa	45 °C	0.5 h	1.04
9		30 MPa	50 °C	1.5 h	2.50
K_1_		7.65	7.06	4.17	-
K_2_		9.54	9.07	9.08	-
K_3_		6.02	7.08	9.96	-
k_1_		2.55	2.35	1.39	-
k_2_		3.18	3.02	3.03	-
k_3_		2.01	2.36	3.32	-
Rj		1.17	0.67	1.96	-

A: Extraction pressure; B: Extraction temperature; C: Extraction time; K: horizontal sum; k: mean value, K/3; Rj: range. The extraction mass was averaged three times.

**Table 3:** Components and peak area percentages of essential oils under different conditions and methods.

NO.	Formula	Categories	Compounds Name	CAS	Area%		
					a	b	c
1	C_9_H_12_O	phenol	mesitol	527-60-6	-	0.82	-
2	C_9_H_14_O	hydrocarbon	4-acetyl-1-methyl-1-cyclohexene	70286-20-3	0.29	-	-
3	C_9_H_14_O	ketone	nopinone	38651-65-9	1.43	-	-
4	C_10_H_16_	hydrocarbon	α-terpinene	99-86-5	-	0.92	-
5	C_10_H_16_	hydrocarbon	2-carene	554-61-0	-	0.53	-
6	C_10_H_16_	hydrocarbon	β-ocimene	502-99-8	-	3.31	-
7	C_10_H_16_	hydrocarbon	β-phellandrene	555-10-2	-	14.77	2.65
8	C_10_H_16_	hydrocarbon	β-pinene	18172-67-3		0.74	-
9	C_10_H_16_	hydrocarbon	α-pinene	7785-26-4	-	4.22	0.72
10	C_10_H_16_O	alcohol	sabinol	471-16-9	0.83	-	-
11	C_10_H_16_O	aldehyde	citral	5392-40-5	-	1.42	-
12	C_10_H_16_O	ketone	camphor	76-22-2	-	3.39	9.05
13	C_10_H_18_O	alcohol	borneol	464-45-9	0.72	0.88	0.62
14	C_10_H_18_O	alcohol	α-terpineol	98-55-5	0.44	4.15	0.47
15	C_10_H_18_O	hydrocarbon	cineole	470-82-6	-	16.37	14.57
16	C_10_H_18_O	alcohol	linalool	78-70-6	15.98	19.81	66.42
17	C_10_H_18_O_2_	oxide	linalool oxide	1365-19-1	-	1.67	0.47
18	C_10_H_20_O	alcohol	citronellol	106-22-9	0.27	-	-
19	C_10_H_10_O_2_	ether	safrole	94-59-7	-	1.06	0.69
20	C_11_H_20_O	alcohol	2-isopropenyl-5-methyl-6-hepten-1-ol	13066-55-2	0.22	-	-
21	C_12_H_20_O_2_	ester	l-bornyl acetate	5655-61-8	0.45	-	-
22	C_14_H_22_O	phenol	2,4-ditert-butyl phenol	96-76-4	-	4.26	0.76
23	C_14_H_28_O	alcohol	9-tetradecen-1-ol	52957-16-1	-	1.37	-
24	C_15_H_24_	hydrocarbon	γ-muurolene	30021-74-0	6.52	-	-
25	C_15_H_24_	hydrocarbon	cubebene	11012-64-9	-	3.55	0.64
26	C_15_H_24_	hydrocarbon	β-caryophyllene	87-44-5	6.60	2.01	-
27	C_15_H_24_	hydrocarbon	α-cubebene	17699-14-8	0.96	-	-
28	C_15_H_24_	hydrocarbon	ylangene	3856-25-5	0.77	-	-
29	C_15_H_24_	hydrocarbon	β-elemene	33880-83-0	1.51	-	-
30	C_15_H_24_	hydrocarbon	humulene	6753-98-6	5.04	-	-
31	C_15_H_24_	hydrocarbon	β-eudesmene	17066-67-0	6.96	-	-
32	C_10_H_18_O	alcohol	terpineol	8000-41-7	-	2.86	1.01
33	C_15_H_22_O	ketone	γ-atlantone	108549-47-9	5.16	-	-
34	C_15_H_22_O	ketone	(*E*)-atlantone	26294-59-7	0.65	-	-
35	C_15_H_24_O	oxide	caryophyllene oxide	1139-30-6	13.07	-	-
36	C_15_H_24_O	oxide	humulene epoxide II	90820-79-4	3.42	-	-
37	C_15_H_26_O	alcohol	nerolidol	7212-44-4	34.59	3.80	0.43
38	C_22_H_46_	hydrocarbon	*n*-docosane	629-97-0	-	2.29	-
39	C_18_H_36_O_2_	acid	stearic acid	57-11-4	-	0.91	-
40	C_19_H_36_O_3_	ester	methyl ricinoleate	23224-20-6	-	2.65	-
41	C_28_H_58_	hydrocarbon	*n*-octacosane	630-02-4	-	1.32	-
42	C_28_H_44_O	alcohol	ergosterol	57-87-4	-	0.69	-

a: Essential oil of camphor tree leaves extracted by steam distillation; b: Essential oil of camphor tree leaves extracted by supercritical CO_2_ extraction at T = 35 °C and P = 15 MPa; c: Essential oil of camphor tree leaves extracted by supercritical CO_2_ extraction at T = 50 °C and P = 30 MPa; Note: “-” is not detected. The average result of the three experiments was taken.

## Data Availability

Not applicable.

## Sample Availability

Samples of the compounds *Cinnamomum camphora* are available from the authors.

## References

[1] Su T., Lin Z.Y., Hou Z.J., Ren Y.M., Liu A.Q. (2021). Research progress of woody plant essential oils. World For. Res..

[2] Barbosa L.C.A., Filomeno C.A., Teixeira R.R. (2016). Chemical Variability and Biological Activities of Eucalyptus spp. Essential Oils. Molecules.

[3] Ghabraie M., Vu K.D., Tata L., Salmieri S., Lacroix M. (2016). Antimicrobial effect of essential oils in combinations against five bacteria and their effect on sensorial quality of ground meat. LWT Food Sci. Technol..

[4] Yang D.C., Zhong C.M., Guo H.Y., Chen Y.L., Zhang L.P., Huang Y.P. (2018). Research Progress on the distribution and control of camphor tree diseases in China. Biol. Disaster Sci..

[5] Chen J.L., Tang C.L., Zhang R.F., Ye S.X., Zhao Z.M., Huang Y.Q., Xu X.J., Lan W.J., Yang D.P. (2020). Metabolomics analysis to evaluate the antibacterial activity of the essential oil from the leaves of Cinnamomum camphora (Linn.) Presl. J. Ethnopharmacol.

[6] Du Y.Q., Zhou H., Yang L.Y., Jiang L.Y., Chen D.F., Qiu D.Y., Yang Y.F. (2022). Advances in Biosynthesis and Pharmacological Effects of *Cinnamomum camphora* (L.) Presl Essential Oil. Forests.

[7] Lahlou M. (2004). Essential oils and fragrance compounds: Bioactivity and mechanisms of action. Flavour Frag. J..

[8] Wu K.G., Lin Y.H., Chai X.H. (2012). Study on gas phase bactericidal mechanism of aromatic camphor oil against *Escherichia coli*. Memb. Conf. Acad. Semin. Guangdong Food Assoc..

[9] Caputo L., Piccialli I., Ciccone R., Caprariis P.D., Pannaccione A. (2020). Lavender and coriander essential oils and their main component linalool exert a protective effect against amyloid-β neurotoxicity. Phytother Res..

[10] Horváth A., Pandur E., Sipos K., Micalizzi G., Mondello L., Böszörményi A., Birinyi P., Horváth G. (2022). Anti-inflammatory effects of lavender and eucalyptus essential oils on the in vitro cell culture model of bladder pain syndrome using T24 cells. BMC Complement. Med..

[11] Santos E.D., Leito M.M., Ito C., Silva-Filho S.E., Kassuya C. (2020). Analgesic and anti-inflammatory articular effects of essential oil and camphor isolated from Ocimum kilimandscharicum Gürke leaves. J. Ethnopharmacol.

[12] Slavko G., Jasenka Č., Andrijana R. (2016). Impact of essential oils on mycelial growth of Botrytis cinerea. Poljoprivreda.

[13] Alonso L., Fernandes K.S., Mendanha S.A., Gonçalves P.J., Gomes R.S., Dorta M.L., Alonso A. (2019). In vitro antileishmanial and cytotoxic activities of nerolidol are associated with changes in plasma membrane dynamics. BBA Biomembr..

[14] Jing C.L., Huang R.H., Su Y., Li Y.Q., Zhang H.S. (2019). Variation in Chemical Composition and Biological Activities of Flos Chrysanthemi indici Essential Oil under Different Extraction Methods. Biomolecules.

[15] Wu F.F., Jin Y.M., Xu X.M., Yang N. (2017). Electrofluidic pretreatment for enhancing essential oil extraction from citrus fruit peel waste. J. Clean Prod..

[16] Zakia B.O.S.S., Hayate H.G., Lila B.M., Peggy R., Hocine R., Abdennour A., Nabyla Khaled K., Khodir M. (2016). Essential oils composition, antibacterial and antioxidant activities of hydrodistillated extract of Eucalyptus globulus fruits. Ind Crop. Prod..

[17] Mohammadhosseini M., Sarker S.D., Akbarzadeh A. (2017). Chemical composition of the essential oils and extracts of Achillea species and their biological activities: A review. J. Ethnopharmacol.

[18] Ferrentino G., Morozova K., Horn C., Scampicchio M. (2020). Extraction of Essential Oils from Medicinal Plants and their Utilization as Food Antioxidants. Curr Pharm Des..

[19] Zermane A., Larkeche O., Meniai A.H., Crampon C., Badens E. (2014). Optimization of essential oil supercritical extraction from Algerian Myrtus communis L. leaves using response surface methodology. J. Supercrit Fluid.

[20] Idris S.A., Rosli N.R., Raja Aris R.M.A. (2022). Supercritical carbon dioxide extraction of fatty acids compounds from tamarind seeds. Mater. Today Proc..

[21] Gaaffar I.F., Zainuddin N.A.M., Zainal S. (2021). Comparison of Identified Compounds from Extracted Pelargonium Radula Leaves by Supercritical Fluid Extraction and Commercial Geranium Essential Oil. IOP Conf. Ser. Mater. Sci. Eng..

[22] Idris S.A., Markom M., Abd Rahman N., Mohd Ali J. (2022). Prediction of overall yield of Gynura procumbens from ethanol-water + supercritical CO_2_ extraction using artificial neural network model. Case Stud. Chem. Environ. Eng..

[23] Zhou Y.P. (2021). Study on Optimization of extraction process of Cinnamomum camphora leaf essential oil by steam distillation. For. Surv. Des..

[24] Zheng H.F., Liao S.L., Fan G.R., Wang Z.D., Chen S.X., Si H.Y. (2019). Extraction of Cinnamomum camphora essential oil by steam distillation and its antibacterial activity. Chem. Ind. For. Prod..

[25] Xiao Z.F., Ai Q., Jin Z.N., Zhang B.H., Wang Y.B., Zhu Y.B. (2021). A study on growth rhythm and dynamic changes of essential oil in *Cinnamomum camphora* dwarf forest. ACTA Agric. Univ. Jiangxiensis.

[26] Goodier M.C., Zhang A.J., Boyd A.H., Hylwa S.A., Goldfarb N.I., Warshaw E.M. (2018). Use of essential oils: A general population survey. Contact Dermat..

[27] Carvalhojr R.N., Corazza M.L., Cardozo-Filho L., Meireles M. (2006). Phase Equilibrium for (Camphor + CO_2_), (Camphor + Propane), and (Camphor + CO_2_ + Propane). J. Chem Eng. Data.

[28] Jokić S., Jerković I., Rajić M., Aladić K., Bilić M., Vidović S. (2017). SC-CO_2_ extraction of Vitex agnus-castus L. fruits: The influence of pressure, temperature and water presoaking on the yield and GC-MS profiles of the extracts in comparison to the essential oil composition. J. Supercrit Fluid.

[29] Yu X.C., Li X.S., Gong Z.W. (2011). Study on extraction of essential oil of camphortree leaves with ultrasonic enhancement of supercritical CO_2_. Chem. Res. Appl..

[30] Guo X.Y. (2019). GC-MS and GC-O analysis of volatile components of Six Edible aromatic plants. J. Agric. Eng..

[31] Plant R.M., Dinh L., Argo S., Shah M. (2019). The Essentials of Essential Oils. Adv. Pediatrics.

[32] Swathi M.G., Ahipa T.N. (2018). Aggregation induced emission properties of new cyanopyridone derivatives. J. Mol. Liq..

[33] Rastakhiz N., Azar P.A., Tehrani M.S., Moradalizadeh M., Larijani K. (2015). Essential oil composition of Artemisia lehmanniana Bunge. Extracted by Hydrodistillation, microwave assisted hydrodistillation and solid phase micro extraction, A comparative study. Int. J. Life Sci..

[34] He F.P., Lei C.Y., Fan J.X., Gong D.Y., Kang Z.M., Liu R., Han S.Q., Luo L.N., Wu X.B., Peng Y. (2019). Research Progress on extraction methods, components and functional properties of plant essential oils. Food Ind. Technol..

[35] Wang W.J., Li R.F. (2016). GC-MS Analysis of the Essential Oil from Ligustrum quihoui Carr. by SFE-CO_2_ and SD Method. J. Anhui Agric. Sci..

[36] Yi H.Y., He G.X., Guo J.S., Pei G. (2010). Comparative study on supercritical CO_2_ extraction and steam distillation extraction of essential oil from Aucklandia. J. Hunan Univ. Tradit. Chin. Med..

[37] Jiang Q.B., Li Q.Y., Wang L., Zhong C.L., Peng Y.H., Wu Y.C. (2018). Analysis of four methods for the extraction of firenuts leaf oil. Mol. Plant Breed..

[38] Luo J., Liu J.Y., Hou Z.H., Chen X., Chu H.J., Zhang Y.Y. (2015). GC-MS analysis of evening primrose oil components extracted by supercritical CO_2_ extraction and water distillation. Preserv. Process..

[39] Donelian A., Carlson L.H.C., Lopes T.J., Machado R.A.F. (2009). Comparison of extraction of patchouli (Pogostemon cablin) essential oil with supercritical CO_2_ and by steam distillation. J. Supercrit Fluid.

[40] Moldão-Martins M., Palavra A., Beirão da Costa M.L., Bernardo-Gil M.G. (2000). Supercritical CO_2_ extraction of *Thymus zygis* L. subsp. *sylvestris* aroma. J. Supercrit Fluid.

[41] Dong J.Y., Liu M.L., Ren B., Zhang M. (2017). Analysis of volatile oil and polysaccharide in Houpo from different habitats. Pharm. Clin. Chin. Mater. Med..

